# [1,3-Bis(diphenyl­phosphan­yl)propane-κ^2^ *P*,*P*′](1,10-phenanthroline-κ^2^ *N*,*N*′)copper(I) perchlorate

**DOI:** 10.1107/S160053681104606X

**Published:** 2011-11-09

**Authors:** Ye-Lan Xiao, Li-Li Zhou, Sen Gao, Qiong-Hua Jin, Cun-Lin Zhang

**Affiliations:** a Department of Chemistry, Capital Normal University, Beijing 100048, People’s Republic of China; b Research Center for Import–Export Chemicals Safety of General Administration of Quality Supervision, Inspection and Quarantine of the People’s Republic of China (AQSIQ), Chinese Academy of Inspection and Quarantine, Beijing 100123, People’s Republic of China; c Key Laboratory of Terahertz Optoelectronics, Ministry of Education, Capital Normal University, Beijing 100048, People’s Republic of China

## Abstract

The title compound, [Cu(C_12_H_8_N_2_)(C_27_H_26_P_2_)]ClO_4_, crystallizes with two Cu^I^ complex cations and two perchlorate anions in the asymmetric unit. Each Cu^I^ cation is four-coordinated by two P atoms of a 1,3-bis­(diphenyl­phosphan­yl)propane mol­ecule and two N atoms of a 1,10-phenanthroline ligand, with a coordination geometry that can be considered as distorted tetra­hedral. The crystal studied was twinned with a twin ratio of 0.786 (2):0.214 (2).

## Related literature

For related structures, see: Abakumov *et al.* (1998 ▶); Saito *et al.* (2006 ▶); Fournier *et al.* (2004 ▶); Affandi *et al.* (1997 ▶); Jin *et al.* (2009 ▶); Alanidis *et al.* (2002 ▶). Potential twinning was indicated by *PLATON* (Spek, 2009 ▶) and confirmed using *ROTAX* (Parson & Gould, 2001 ▶) as included in the *WinGX* suite (Farrugia, 1999 ▶). 
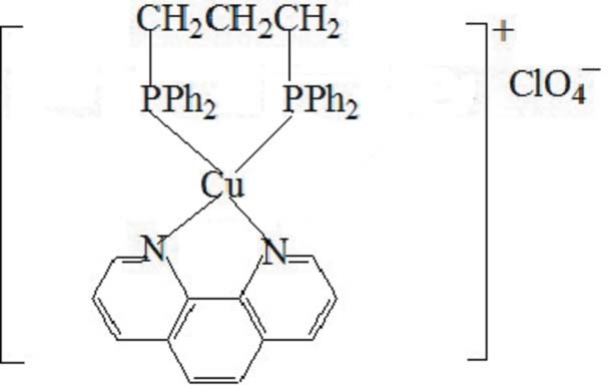


## Experimental

### 

#### Crystal data

[Cu(C_12_H_8_N_2_)(C_27_H_26_P_2_)]ClO_4_
*M* *_r_* = 755.61
Monoclinic, 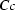

*a* = 16.6548 (15) Å
*b* = 12.9238 (12) Å
*c* = 32.936 (3) Å
β = 90.126 (1)°
*V* = 7089.2 (11) Å^3^
*Z* = 8
Mo *K*α radiation
μ = 0.83 mm^−1^
*T* = 298 K
0.45 × 0.40 × 0.20 mm

#### Data collection

Bruker SMART CCD area-detector diffractometer
Absorption correction: multi-scan (*SADABS*; Sheldrick, 1996 ▶) *T*_min_ = 0.708, *T*_max_ = 0.852
10139 measured reflections
10139 independent reflections
8324 reflections with *I* > 2σ(*I*)
*R*_int_ = 0.035

#### Refinement

*R*[*F* ^2^ > 2σ(*F* ^2^)] = 0.038
*wR*(*F* ^2^) = 0.074
*S* = 0.91
10139 reflections
884 parameters
2 restraints
H-atom parameters constrained
Δρ_max_ = 0.34 e Å^−3^
Δρ_min_ = −0.27 e Å^−3^
Absolute structure: Flack (1983 ▶), 6243 Friedel pairs
Flack parameter: −0.009 (12)

### 

Data collection: *SMART* (Bruker, 2007 ▶); cell refinement: *SAINT* (Bruker, 2007 ▶); data reduction: *SAINT*; program(s) used to solve structure: *SHELXS97* (Sheldrick, 2008 ▶); program(s) used to refine structure: *SHELXL97* (Sheldrick, 2008 ▶); molecular graphics: *SHELXTL* (Sheldrick, 2008 ▶) and *Mercury* (Macrae *et al.*, 2008 ▶); software used to prepare material for publication: *SHELXTL*.

## Supplementary Material

Crystal structure: contains datablock(s) global, I. DOI: 10.1107/S160053681104606X/lr2032sup1.cif

Structure factors: contains datablock(s) I. DOI: 10.1107/S160053681104606X/lr2032Isup2.hkl

Additional supplementary materials:  crystallographic information; 3D view; checkCIF report

## Figures and Tables

**Fig. 1.**
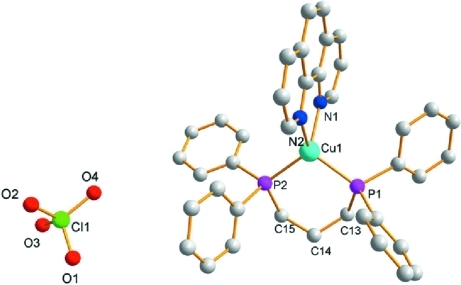
A view of the structure of one of the two geometrically equivalent CuI complex and perchlorate moieties present in the asymmetric unit. Atoms are displayed as elliposoids at the 50% probability level

## supplementary crystallographic information

### Comment

There has been an increasing interest in the photophysics and photochemistry of Cu^I^ complexes in the last few decades. Especially Cu^I^complexes of mixed ligand phosphines and 1,10-phenanthroline (phen) have been often used as interesting emitters. Among the diphosphine ligands known in the literature, 1,3-Bis(diphenylphosphanyl) propane (dppp) is extensively studied since dppp is a very efficient bidentate ligand to lock one metal atom (Abakumov *et al.*,1998;Saito *et al.*,2006;Fournier *et al.*,2004; Affandi *et al.*,1997). As a part of the extension of our study on photophysical properties of copper(I) complexes with ligands containing phosphine and nitrogen atoms(Jin *et al.*, 2009), we synthesized a new complex with the formula [Cu(dppp)(phen)] ^+^ (ClO_4_)^-^ .

The bond distances, bond and torsion angles in the two Cu^I^ complex moieties present in the asymmetric units are quite similar, in fact the two molecules can be almost perfectly overlayed, *Mercury* (Macrae *et al.*, 2008). Each copper(I) cation is four-coordinated by two phosphorus atoms from dppp, and two nitrogen atoms from phen. The angles P—Cu—P, N—P—N and P—Cu—N are in the range of 104.71 (12)°-105.03 (12)°, 81.0 (4)°-81.2 (4) and 108.7 (3)°-124.1 (3)°, respectively, which confirms the distorted tetrahedral environment around both of the copper(I) cations (Fig. 1).

### Experimental

A mixture of Cu(ClO_4_)_2_(55.58 mg, 0.15 mmol) and Cu (9.53 mg, 0.15 mmol) were stirred in a solution of CH_3_CN(15 ml) and CH_2_Cl_2_(5 ml), after half one hour the blue solution became colorless,and then 1,10-phen (39.6 mg, 0.3 mmol) and dppp (123.73 mg, 0.3 mmol) were added and the solution stirred for 3 h. The yellow filtrate was allowed to stand at room temperature for several days and yellow crystal of the title complex [Cu(dppp)(phen)](ClO_4_) were obtained. Crystals suitable for single-crystal X-ray diffraction were selected directly from the sample as prepared.

### Refinement

Metal atom were located from the E-maps and other non-hydrogen atoms were located in successive difference Fourier syntheses. H atoms were geometrically positioned and refined in the riding model approximation, with C—H =0.93 and 0.97 Å for aromatic and methylene respectively and U_iso_(H)=1.2U_eq_(C).

Potential twinning was indicates by  *PLATON* (Spek, 2009) and confirmed using *ROTAX* program (Parson & Gould, 2001) as included in the *WINGX* suite (Farrugia, 1999). Refinements were done using the twining matrix [-1 0 0, 0 -1 0, 0 0 1], which gave a twin ratio of 0.786:0.214 (2).

### Crystal data

**Table:**

[Cu(C_12_H_8_N_2_)(C_27_H_26_P_2_)]ClO_4_	*F*(000) = 3120
*M**_r_* = 755.61	*D*_x_ = 1.417 Mg m^−^^3^
Monoclinic, *C**c*	Mo *K*α radiation, λ = 0.71073 Å
Hall symbol: C -2yc	Cell parameters from 6082 reflections
*a* = 16.6548 (15) Å	θ = 2.7–23.6°
*b* = 12.9238 (12) Å	µ = 0.83 mm^−^^1^
*c* = 32.936 (3) Å	*T* = 298 K
β = 90.126 (1)°	Block, yellow
*V* = 7089.2 (11) Å^3^	0.45 × 0.40 × 0.20 mm
*Z* = 8	

### Data collection

**Table:**

Bruker SMART CCD area-detector diffractometer	10139 independent reflections
Radiation source: fine-focus sealed tube	8324 reflections with *I* > 2σ(*I*)
graphite	*R*_int_ = 0.035
phi and ω scans	θ_max_ = 25.0°, θ_min_ = 2.4°
Absorption correction: multi-scan (*SADABS*; Sheldrick, 1996)	*h* = −19→19
*T*_min_ = 0.708, *T*_max_ = 0.852	*k* = −15→12
10139 measured reflections	*l* = −39→29

### Refinement

**Table:**

Refinement on *F*^2^	Secondary atom site location: difference Fourier map
Least-squares matrix: full	Hydrogen site location: inferred from neighbouring sites
*R*[*F*^2^ > 2σ(*F*^2^)] = 0.038	H-atom parameters constrained
*wR*(*F*^2^) = 0.074	*w* = 1/[σ^2^(*F*_o_^2^) + (0.0325*P*)^2^] where *P* = (*F*_o_^2^ + 2*F*_c_^2^)/3
*S* = 0.91	(Δ/σ)_max_ < 0.001
10139 reflections	Δρ_max_ = 0.34 e Å^−^^3^
884 parameters	Δρ_min_ = −0.27 e Å^−^^3^
2 restraints	Absolute structure: Flack (1983), 6243 Friedel pairs
Primary atom site location: structure-invariant direct methods	Flack parameter: −0.009 (12)

### Special details

**Table:**

Geometry. All esds (except the esd in the dihedral angle between two l.s. planes) are estimated using the full covariance matrix. The cell esds are taken into account individually in the estimation of esds in distances, angles and torsion angles; correlations between esds in cell parameters are only used when they are defined by crystal symmetry. An approximate (isotropic) treatment of cell esds is used for estimating esds involving l.s. planes.
Refinement. Refinement of *F*^2^ against ALL reflections. The weighted *R*-factor *wR* and goodness of fit *S* are based on *F*^2^, conventional *R*-factors *R* are based on *F*, with *F* set to zero for negative *F*^2^. The threshold expression of *F*^2^ > σ(*F*^2^) is used only for calculating *R*-factors(gt) *etc*. and is not relevant to the choice of reflections for refinement. *R*-factors based on *F*^2^ are statistically about twice as large as those based on *F*, and *R*- factors based on ALL data will be even larger.

### Fractional atomic coordinates and isotropic or equivalent isotropic displacement parameters (Å^2^)

**Table:**

	*x*	*y*	*z*	*U*_iso_*/*U*_eq_	
Cu1	0.42671 (4)	0.72241 (5)	0.39790 (2)	0.03690 (17)	
P1	0.47763 (8)	0.69379 (10)	0.45975 (5)	0.0357 (3)	
P2	0.53343 (9)	0.75548 (11)	0.35901 (5)	0.0358 (3)	
N1	0.3358 (2)	0.8312 (3)	0.40279 (14)	0.0331 (11)	
N2	0.3362 (2)	0.6372 (3)	0.37241 (14)	0.0364 (11)	
C1	0.3335 (3)	0.9226 (4)	0.42048 (19)	0.0427 (15)	
H1	0.3817	0.9498	0.4301	0.051*	
C2	0.2656 (4)	0.9803 (4)	0.4258 (2)	0.0503 (16)	
H2	0.2684	1.0449	0.4381	0.060*	
C3	0.1945 (4)	0.9425 (5)	0.4130 (2)	0.0557 (18)	
H3	0.1475	0.9796	0.4175	0.067*	
C4	0.1917 (3)	0.8466 (4)	0.3927 (2)	0.0436 (15)	
C5	0.2645 (3)	0.7929 (4)	0.38937 (17)	0.0321 (13)	
C6	0.2653 (3)	0.6905 (4)	0.37148 (16)	0.0293 (12)	
C7	0.1941 (3)	0.6499 (4)	0.35579 (19)	0.0421 (14)	
C8	0.1975 (4)	0.5513 (5)	0.33929 (19)	0.0517 (17)	
H8	0.1520	0.5224	0.3274	0.062*	
C9	0.2677 (4)	0.4965 (5)	0.34041 (19)	0.0537 (17)	
H9	0.2705	0.4299	0.3298	0.064*	
C10	0.3341 (3)	0.5427 (4)	0.3576 (2)	0.0458 (15)	
H10	0.3811	0.5040	0.3589	0.055*	
C11	0.1204 (4)	0.8038 (6)	0.3764 (2)	0.0555 (18)	
H11	0.0723	0.8401	0.3782	0.067*	
C12	0.1220 (4)	0.7101 (5)	0.3581 (2)	0.0579 (18)	
H12	0.0750	0.6840	0.3468	0.069*	
C13	0.5725 (3)	0.7652 (4)	0.46446 (18)	0.0426 (15)	
H13A	0.5612	0.8388	0.4644	0.051*	
H13B	0.5973	0.7482	0.4903	0.051*	
C14	0.6319 (3)	0.7406 (4)	0.43006 (18)	0.0445 (15)	
H14A	0.6290	0.6672	0.4242	0.053*	
H14B	0.6858	0.7552	0.4397	0.053*	
C15	0.6180 (3)	0.8006 (4)	0.39041 (19)	0.0405 (15)	
H15A	0.6666	0.7968	0.3743	0.049*	
H15B	0.6092	0.8728	0.3971	0.049*	
C16	0.5047 (3)	0.5636 (4)	0.47674 (18)	0.0370 (13)	
C17	0.5514 (4)	0.5449 (5)	0.5100 (2)	0.0589 (19)	
H17	0.5710	0.6006	0.5249	0.071*	
C18	0.5701 (5)	0.4464 (6)	0.5221 (2)	0.073 (2)	
H18	0.6006	0.4356	0.5454	0.087*	
C19	0.5437 (4)	0.3635 (5)	0.4995 (2)	0.065 (2)	
H19	0.5585	0.2964	0.5066	0.077*	
C20	0.4961 (4)	0.3805 (5)	0.4671 (2)	0.066 (2)	
H20	0.4764	0.3244	0.4525	0.079*	
C21	0.4760 (4)	0.4798 (4)	0.4552 (2)	0.0502 (16)	
H21	0.4431	0.4902	0.4327	0.060*	
C22	0.4171 (4)	0.7430 (4)	0.50179 (19)	0.0451 (15)	
C23	0.4487 (5)	0.7817 (6)	0.5368 (2)	0.081 (2)	
H23	0.5039	0.7825	0.5409	0.097*	
C24	0.3961 (7)	0.8204 (8)	0.5666 (3)	0.111 (3)	
H24	0.4178	0.8468	0.5905	0.133*	
C25	0.3168 (7)	0.8208 (6)	0.5621 (3)	0.092 (3)	
H25	0.2836	0.8474	0.5822	0.111*	
C26	0.2863 (5)	0.7821 (6)	0.5281 (3)	0.074 (2)	
H26	0.2308	0.7805	0.5247	0.088*	
C27	0.3352 (4)	0.7439 (5)	0.4974 (2)	0.0580 (18)	
H27	0.3121	0.7187	0.4737	0.070*	
C28	0.5713 (3)	0.6449 (4)	0.33030 (18)	0.0392 (14)	
C29	0.6472 (3)	0.6057 (5)	0.33432 (19)	0.0494 (16)	
H29	0.6827	0.6345	0.3530	0.059*	
C30	0.6710 (4)	0.5227 (6)	0.3103 (2)	0.0642 (19)	
H30	0.7222	0.4954	0.3137	0.077*	
C31	0.6204 (5)	0.4803 (5)	0.2818 (3)	0.075 (2)	
H31	0.6376	0.4259	0.2655	0.090*	
C32	0.5451 (5)	0.5189 (6)	0.2779 (3)	0.080 (2)	
H32	0.5104	0.4906	0.2587	0.096*	
C33	0.5195 (4)	0.5999 (5)	0.3019 (2)	0.0556 (17)	
H33	0.4673	0.6247	0.2992	0.067*	
C34	0.5288 (3)	0.8567 (4)	0.32093 (17)	0.0371 (13)	
C35	0.4785 (4)	0.9415 (5)	0.3279 (2)	0.0510 (16)	
H35	0.4474	0.9435	0.3513	0.061*	
C36	0.4743 (4)	1.0228 (5)	0.3005 (2)	0.0625 (19)	
H36	0.4406	1.0788	0.3055	0.075*	
C37	0.5199 (5)	1.0202 (6)	0.2660 (2)	0.065 (2)	
H37	0.5184	1.0755	0.2480	0.077*	
C38	0.5679 (5)	0.9367 (6)	0.2580 (2)	0.066 (2)	
H38	0.5973	0.9347	0.2341	0.079*	
C39	0.5731 (4)	0.8553 (5)	0.28510 (19)	0.0485 (16)	
H39	0.6063	0.7993	0.2794	0.058*	
Cu2	0.67768 (4)	0.02367 (5)	0.12969 (2)	0.03601 (16)	
P3	0.78350 (8)	−0.00721 (11)	0.16949 (5)	0.0354 (3)	
P4	0.73055 (8)	0.05612 (10)	0.06834 (5)	0.0343 (3)	
N3	0.5868 (2)	0.1137 (3)	0.15442 (14)	0.0351 (11)	
N4	0.5858 (2)	−0.0825 (3)	0.12632 (14)	0.0336 (11)	
C40	0.5885 (3)	0.2093 (4)	0.16869 (18)	0.0414 (14)	
H40	0.6370	0.2450	0.1685	0.050*	
C41	0.5204 (4)	0.2587 (4)	0.1840 (2)	0.0505 (16)	
H41	0.5238	0.3258	0.1940	0.061*	
C42	0.4494 (4)	0.2076 (5)	0.18395 (19)	0.0511 (17)	
H42	0.4037	0.2399	0.1941	0.061*	
C43	0.4444 (3)	0.1067 (4)	0.16880 (18)	0.0380 (14)	
C44	0.5157 (3)	0.0626 (4)	0.15437 (16)	0.0302 (12)	
C45	0.5148 (3)	−0.0407 (4)	0.13846 (16)	0.0301 (13)	
C46	0.4417 (3)	−0.0937 (4)	0.13530 (19)	0.0428 (15)	
C47	0.4435 (4)	−0.1931 (5)	0.1176 (2)	0.0525 (17)	
H47	0.3963	−0.2305	0.1140	0.063*	
C48	0.5143 (4)	−0.2330 (5)	0.1061 (2)	0.0564 (18)	
H48	0.5163	−0.2991	0.0949	0.068*	
C49	0.5841 (3)	−0.1769 (4)	0.11057 (18)	0.0440 (15)	
H49	0.6321	−0.2066	0.1022	0.053*	
C50	0.3722 (4)	0.0494 (5)	0.1668 (2)	0.0542 (18)	
H50	0.3252	0.0776	0.1773	0.065*	
C51	0.3705 (4)	−0.0462 (5)	0.1498 (2)	0.0545 (18)	
H51	0.3219	−0.0811	0.1475	0.065*	
C52	0.8703 (3)	−0.0452 (4)	0.13890 (17)	0.0394 (15)	
H52A	0.9180	−0.0384	0.1556	0.047*	
H52B	0.8646	−0.1179	0.1321	0.047*	
C53	0.8841 (3)	0.0152 (5)	0.09922 (18)	0.0445 (15)	
H53A	0.8793	0.0886	0.1050	0.053*	
H53B	0.9387	0.0027	0.0902	0.053*	
C54	0.8272 (3)	−0.0116 (4)	0.06470 (18)	0.0422 (15)	
H54A	0.8176	−0.0856	0.0648	0.051*	
H54B	0.8524	0.0061	0.0391	0.051*	
C55	0.8147 (3)	0.1043 (4)	0.19919 (18)	0.0423 (14)	
C56	0.8901 (4)	0.1481 (6)	0.1967 (2)	0.068 (2)	
H56	0.9282	0.1203	0.1792	0.081*	
C57	0.9094 (6)	0.2339 (7)	0.2204 (3)	0.091 (3)	
H57	0.9602	0.2632	0.2188	0.109*	
C58	0.8531 (8)	0.2749 (7)	0.2460 (3)	0.103 (4)	
H58	0.8658	0.3330	0.2614	0.124*	
C59	0.7787 (6)	0.2317 (6)	0.2492 (3)	0.093 (3)	
H59	0.7414	0.2596	0.2671	0.112*	
C60	0.7588 (4)	0.1459 (5)	0.2258 (2)	0.064 (2)	
H60	0.7080	0.1164	0.2279	0.077*	
C61	0.7826 (3)	−0.1098 (4)	0.20750 (18)	0.0385 (14)	
C62	0.8327 (4)	−0.1084 (5)	0.24078 (19)	0.0491 (16)	
H62	0.8650	−0.0508	0.2453	0.059*	
C63	0.8363 (5)	−0.1901 (6)	0.2676 (2)	0.067 (2)	
H63	0.8710	−0.1879	0.2898	0.080*	
C64	0.7881 (5)	−0.2751 (6)	0.2614 (2)	0.068 (2)	
H64	0.7903	−0.3308	0.2793	0.081*	
C65	0.7364 (4)	−0.2776 (5)	0.2286 (2)	0.0592 (18)	
H65	0.7033	−0.3346	0.2246	0.071*	
C66	0.7341 (3)	−0.1953 (4)	0.2019 (2)	0.0488 (15)	
H66	0.6993	−0.1975	0.1798	0.059*	
C67	0.7561 (3)	0.1873 (4)	0.05374 (17)	0.0358 (13)	
C68	0.7223 (3)	0.2701 (4)	0.0740 (2)	0.0491 (16)	
H68	0.6863	0.2576	0.0950	0.059*	
C69	0.7408 (4)	0.3720 (5)	0.0637 (2)	0.0631 (19)	
H69	0.7184	0.4269	0.0780	0.076*	
C70	0.7919 (5)	0.3897 (5)	0.0327 (2)	0.070 (2)	
H70	0.8038	0.4576	0.0255	0.084*	
C71	0.8261 (5)	0.3114 (6)	0.0119 (2)	0.076 (2)	
H71	0.8617	0.3256	−0.0091	0.091*	
C72	0.8081 (4)	0.2100 (5)	0.0217 (2)	0.0622 (19)	
H72	0.8308	0.1563	0.0068	0.075*	
C73	0.6754 (4)	0.0081 (4)	0.02457 (19)	0.0439 (15)	
C74	0.5942 (4)	−0.0083 (5)	0.0283 (2)	0.065 (2)	
H74	0.5687	0.0070	0.0526	0.078*	
C75	0.5490 (6)	−0.0481 (7)	−0.0045 (3)	0.093 (3)	
H75	0.4943	−0.0602	−0.0016	0.112*	
C76	0.5865 (8)	−0.0689 (6)	−0.0407 (4)	0.110 (4)	
H76	0.5574	−0.0958	−0.0624	0.132*	
C77	0.6654 (6)	−0.0500 (7)	−0.0445 (3)	0.108 (4)	
H77	0.6905	−0.0616	−0.0692	0.129*	
C78	0.7092 (5)	−0.0140 (6)	−0.0124 (2)	0.082 (3)	
H78	0.7641	−0.0041	−0.0157	0.099*	
Cl1	0.62460 (9)	0.52007 (15)	0.14978 (6)	0.0629 (5)	
O1	0.6741 (3)	0.4530 (4)	0.1715 (2)	0.100 (2)	
O2	0.5784 (3)	0.4606 (6)	0.1222 (2)	0.123 (3)	
O3	0.6722 (4)	0.5902 (4)	0.1281 (2)	0.107 (2)	
O4	0.5722 (4)	0.5705 (7)	0.1754 (2)	0.158 (3)	
Cl2	0.37682 (10)	0.22630 (15)	0.38519 (5)	0.0607 (5)	
O5	0.4267 (3)	0.2954 (4)	0.36355 (18)	0.0923 (18)	
O6	0.3291 (4)	0.1696 (6)	0.3587 (2)	0.140 (3)	
O7	0.4233 (3)	0.1602 (4)	0.41025 (18)	0.0929 (19)	
O8	0.3269 (4)	0.2849 (6)	0.4107 (3)	0.143 (3)	

### Atomic displacement parameters (Å^2^)

**Table:**

	*U*^11^	*U*^22^	*U*^33^	*U*^12^	*U*^13^	*U*^23^
Cu1	0.0283 (3)	0.0425 (4)	0.0398 (4)	−0.0013 (3)	−0.0002 (3)	−0.0015 (4)
P1	0.0346 (8)	0.0400 (8)	0.0326 (9)	−0.0008 (6)	0.0026 (7)	−0.0021 (7)
P2	0.0309 (7)	0.0416 (9)	0.0349 (9)	−0.0024 (6)	0.0016 (7)	0.0027 (7)
N1	0.033 (3)	0.030 (2)	0.036 (3)	−0.0017 (19)	−0.001 (2)	−0.002 (2)
N2	0.034 (3)	0.036 (3)	0.038 (3)	0.002 (2)	0.003 (2)	−0.005 (2)
C1	0.041 (3)	0.036 (3)	0.051 (4)	−0.003 (3)	−0.004 (3)	−0.002 (3)
C2	0.059 (4)	0.033 (3)	0.059 (4)	0.005 (3)	0.009 (3)	−0.006 (3)
C3	0.044 (4)	0.043 (4)	0.080 (5)	0.020 (3)	0.004 (4)	0.005 (3)
C4	0.033 (3)	0.046 (4)	0.052 (4)	0.010 (3)	0.003 (3)	0.010 (3)
C5	0.034 (3)	0.027 (3)	0.036 (4)	−0.004 (2)	0.003 (3)	0.012 (2)
C6	0.025 (3)	0.039 (3)	0.023 (3)	−0.006 (2)	0.000 (2)	0.007 (2)
C7	0.037 (3)	0.047 (4)	0.042 (4)	−0.011 (3)	0.003 (3)	0.005 (3)
C8	0.044 (4)	0.065 (5)	0.046 (4)	−0.024 (3)	−0.007 (3)	−0.007 (3)
C9	0.068 (5)	0.045 (4)	0.048 (4)	−0.014 (3)	0.006 (4)	−0.015 (3)
C10	0.046 (4)	0.032 (3)	0.060 (4)	−0.002 (3)	0.010 (3)	−0.005 (3)
C11	0.032 (3)	0.071 (5)	0.063 (5)	0.000 (3)	−0.001 (3)	0.010 (4)
C12	0.035 (3)	0.074 (5)	0.065 (5)	−0.010 (3)	−0.019 (3)	0.010 (4)
C13	0.051 (4)	0.042 (3)	0.035 (4)	−0.007 (3)	0.000 (3)	−0.010 (3)
C14	0.038 (3)	0.051 (4)	0.045 (4)	−0.005 (3)	−0.009 (3)	0.004 (3)
C15	0.032 (3)	0.044 (4)	0.046 (4)	−0.002 (3)	0.003 (3)	0.000 (3)
C16	0.032 (3)	0.041 (3)	0.038 (4)	−0.005 (2)	0.003 (3)	−0.009 (3)
C17	0.079 (5)	0.044 (4)	0.054 (5)	−0.001 (3)	−0.023 (4)	−0.003 (3)
C18	0.095 (6)	0.067 (5)	0.056 (5)	0.003 (4)	−0.019 (4)	0.014 (4)
C19	0.075 (5)	0.035 (4)	0.084 (6)	0.003 (3)	0.003 (4)	0.011 (4)
C20	0.076 (5)	0.050 (4)	0.072 (6)	−0.011 (4)	−0.002 (4)	−0.010 (4)
C21	0.056 (4)	0.048 (4)	0.047 (4)	−0.009 (3)	−0.007 (3)	0.001 (3)
C22	0.056 (4)	0.037 (3)	0.042 (4)	−0.005 (3)	0.013 (3)	0.002 (3)
C23	0.070 (5)	0.113 (7)	0.061 (5)	−0.016 (5)	0.014 (4)	−0.040 (5)
C24	0.134 (9)	0.134 (8)	0.065 (6)	−0.023 (7)	0.030 (6)	−0.054 (6)
C25	0.126 (8)	0.071 (5)	0.079 (7)	0.008 (5)	0.066 (7)	−0.011 (5)
C26	0.078 (5)	0.069 (5)	0.074 (6)	0.028 (4)	0.033 (5)	0.023 (5)
C27	0.062 (4)	0.064 (4)	0.048 (4)	0.023 (3)	0.017 (4)	0.020 (3)
C28	0.032 (3)	0.041 (3)	0.044 (4)	0.000 (2)	−0.001 (3)	0.007 (3)
C29	0.044 (4)	0.063 (4)	0.041 (4)	0.012 (3)	0.006 (3)	0.000 (3)
C30	0.055 (4)	0.073 (5)	0.064 (5)	0.031 (4)	0.010 (4)	0.004 (4)
C31	0.089 (6)	0.058 (5)	0.078 (6)	0.019 (4)	0.018 (5)	−0.012 (4)
C32	0.077 (5)	0.079 (5)	0.083 (6)	0.002 (4)	−0.007 (5)	−0.037 (5)
C33	0.043 (4)	0.058 (4)	0.066 (5)	0.001 (3)	−0.004 (3)	−0.014 (3)
C34	0.031 (3)	0.050 (4)	0.030 (3)	0.003 (3)	−0.004 (3)	−0.002 (3)
C35	0.044 (3)	0.058 (4)	0.051 (4)	0.002 (3)	0.002 (3)	0.011 (3)
C36	0.060 (4)	0.054 (4)	0.073 (6)	0.011 (3)	−0.014 (4)	0.014 (4)
C37	0.086 (5)	0.054 (5)	0.054 (5)	−0.008 (4)	−0.021 (4)	0.019 (4)
C38	0.092 (5)	0.069 (5)	0.036 (4)	−0.020 (4)	−0.001 (4)	0.014 (4)
C39	0.051 (4)	0.051 (4)	0.044 (4)	0.000 (3)	0.002 (3)	−0.003 (3)
Cu2	0.0270 (3)	0.0389 (4)	0.0421 (4)	0.0016 (3)	0.0033 (3)	−0.0024 (4)
P3	0.0292 (7)	0.0399 (9)	0.0371 (9)	0.0026 (6)	0.0015 (7)	0.0000 (7)
P4	0.0314 (8)	0.0373 (8)	0.0342 (9)	0.0001 (6)	0.0001 (7)	−0.0036 (6)
N3	0.030 (3)	0.035 (3)	0.040 (3)	0.0040 (19)	0.006 (2)	−0.001 (2)
N4	0.034 (3)	0.029 (2)	0.038 (3)	0.0022 (19)	0.006 (2)	−0.002 (2)
C40	0.041 (3)	0.038 (3)	0.046 (4)	−0.001 (3)	−0.001 (3)	−0.003 (3)
C41	0.060 (4)	0.030 (3)	0.061 (5)	0.015 (3)	0.004 (4)	−0.007 (3)
C42	0.044 (4)	0.062 (4)	0.048 (4)	0.023 (3)	0.015 (3)	0.004 (3)
C43	0.034 (3)	0.043 (3)	0.037 (4)	0.015 (3)	0.008 (3)	0.003 (3)
C44	0.027 (3)	0.034 (3)	0.030 (3)	0.002 (2)	0.000 (2)	0.003 (2)
C45	0.028 (3)	0.033 (3)	0.030 (3)	0.002 (2)	0.007 (3)	0.006 (2)
C46	0.035 (3)	0.043 (4)	0.051 (4)	−0.003 (3)	0.000 (3)	0.008 (3)
C47	0.039 (4)	0.052 (4)	0.066 (5)	−0.018 (3)	0.004 (3)	0.000 (3)
C48	0.061 (4)	0.038 (3)	0.070 (5)	−0.011 (3)	−0.005 (4)	−0.015 (3)
C49	0.041 (3)	0.037 (3)	0.053 (4)	0.000 (3)	0.003 (3)	−0.011 (3)
C50	0.032 (3)	0.070 (5)	0.060 (5)	0.013 (3)	0.004 (3)	0.005 (4)
C51	0.031 (4)	0.067 (5)	0.065 (5)	−0.009 (3)	0.000 (3)	0.008 (4)
C52	0.035 (3)	0.050 (4)	0.033 (4)	0.003 (2)	−0.011 (3)	0.004 (3)
C53	0.029 (3)	0.066 (4)	0.038 (4)	0.007 (3)	0.006 (3)	0.003 (3)
C54	0.045 (3)	0.048 (4)	0.034 (4)	0.008 (3)	0.002 (3)	0.001 (3)
C55	0.043 (4)	0.045 (3)	0.040 (4)	−0.004 (3)	−0.011 (3)	0.002 (3)
C56	0.068 (5)	0.081 (5)	0.054 (5)	−0.021 (4)	−0.003 (4)	0.004 (4)
C57	0.104 (8)	0.092 (6)	0.077 (6)	−0.048 (6)	−0.025 (6)	−0.007 (6)
C58	0.174 (11)	0.063 (6)	0.073 (7)	−0.029 (7)	−0.056 (7)	−0.012 (5)
C59	0.132 (8)	0.060 (5)	0.088 (7)	0.028 (6)	−0.030 (6)	−0.022 (5)
C60	0.066 (5)	0.059 (4)	0.068 (5)	0.013 (4)	−0.009 (4)	−0.016 (4)
C61	0.037 (3)	0.039 (3)	0.040 (4)	0.013 (3)	0.009 (3)	−0.003 (3)
C62	0.059 (4)	0.048 (4)	0.040 (4)	0.015 (3)	−0.002 (3)	−0.003 (3)
C63	0.091 (6)	0.070 (5)	0.039 (4)	0.025 (4)	0.004 (4)	0.001 (4)
C64	0.083 (5)	0.074 (5)	0.046 (5)	0.026 (4)	0.025 (4)	0.020 (4)
C65	0.062 (4)	0.047 (4)	0.069 (5)	0.004 (3)	0.025 (4)	0.014 (4)
C66	0.042 (3)	0.054 (4)	0.051 (4)	0.008 (3)	0.001 (3)	0.003 (3)
C67	0.035 (3)	0.037 (3)	0.035 (3)	−0.003 (2)	−0.006 (3)	−0.002 (2)
C68	0.050 (4)	0.043 (4)	0.054 (4)	0.007 (3)	0.007 (3)	0.001 (3)
C69	0.088 (5)	0.042 (4)	0.060 (5)	0.010 (4)	0.008 (4)	−0.005 (3)
C70	0.101 (6)	0.047 (4)	0.062 (5)	−0.007 (4)	−0.004 (5)	0.009 (4)
C71	0.108 (6)	0.057 (5)	0.063 (5)	−0.012 (4)	0.035 (5)	0.001 (4)
C72	0.080 (5)	0.049 (4)	0.057 (5)	−0.008 (4)	0.029 (4)	−0.002 (3)
C73	0.051 (4)	0.034 (3)	0.047 (4)	0.004 (3)	−0.012 (3)	0.000 (3)
C74	0.068 (5)	0.072 (5)	0.056 (5)	−0.021 (4)	−0.013 (4)	0.018 (4)
C75	0.088 (6)	0.081 (6)	0.110 (8)	−0.035 (5)	−0.062 (6)	0.022 (6)
C76	0.142 (10)	0.063 (5)	0.126 (10)	0.011 (6)	−0.089 (9)	−0.038 (6)
C77	0.130 (9)	0.116 (7)	0.077 (6)	0.057 (7)	−0.051 (7)	−0.053 (5)
C78	0.072 (5)	0.116 (7)	0.059 (5)	0.028 (5)	−0.018 (5)	−0.033 (5)
Cl1	0.0366 (9)	0.0854 (12)	0.0668 (12)	0.0051 (9)	0.0085 (9)	0.0126 (10)
O1	0.057 (3)	0.109 (4)	0.135 (5)	0.005 (3)	−0.022 (4)	0.045 (4)
O2	0.078 (4)	0.180 (7)	0.112 (6)	−0.049 (4)	−0.034 (4)	0.016 (5)
O3	0.088 (4)	0.074 (3)	0.159 (6)	0.000 (3)	0.036 (4)	0.036 (4)
O4	0.126 (6)	0.231 (9)	0.118 (6)	0.085 (6)	0.056 (5)	0.002 (6)
Cl2	0.0373 (9)	0.0942 (13)	0.0507 (11)	−0.0079 (9)	−0.0045 (8)	0.0133 (10)
O5	0.048 (3)	0.132 (5)	0.096 (4)	−0.019 (3)	0.001 (3)	0.042 (4)
O6	0.120 (5)	0.199 (7)	0.101 (6)	−0.088 (5)	−0.054 (5)	0.025 (5)
O7	0.090 (4)	0.084 (3)	0.104 (5)	−0.013 (3)	−0.039 (4)	0.023 (3)
O8	0.082 (5)	0.202 (7)	0.146 (7)	0.051 (5)	0.053 (5)	0.027 (6)

### Geometric parameters (Å, °)

**Table:**

Cu1—N2	2.046 (4)	P3—C55	1.817 (6)
Cu1—N1	2.073 (4)	P3—C61	1.823 (6)
Cu1—P2	2.2344 (16)	P3—C52	1.831 (6)
Cu1—P1	2.2355 (16)	P4—C67	1.813 (5)
P1—C16	1.830 (6)	P4—C73	1.817 (6)
P1—C22	1.829 (6)	P4—C54	1.836 (5)
P1—C13	1.836 (5)	N3—C40	1.322 (6)
P2—C34	1.813 (6)	N3—C44	1.355 (6)
P2—C28	1.826 (6)	N4—C49	1.327 (6)
P2—C15	1.841 (6)	N4—C45	1.361 (6)
N1—C1	1.317 (7)	C40—C41	1.396 (8)
N1—C5	1.360 (6)	C40—H40	0.9300
N2—C10	1.315 (6)	C41—C42	1.355 (8)
N2—C6	1.367 (6)	C41—H41	0.9300
C1—C2	1.367 (7)	C42—C43	1.399 (8)
C1—H1	0.9300	C42—H42	0.9300
C2—C3	1.347 (8)	C43—C44	1.403 (7)
C2—H2	0.9300	C43—C50	1.413 (8)
C3—C4	1.408 (8)	C44—C45	1.433 (7)
C3—H3	0.9300	C45—C46	1.401 (7)
C4—C5	1.402 (7)	C46—C47	1.411 (8)
C4—C11	1.417 (8)	C46—C51	1.418 (8)
C5—C6	1.449 (7)	C47—C48	1.343 (8)
C6—C7	1.395 (7)	C47—H47	0.9300
C7—C8	1.387 (8)	C48—C49	1.377 (7)
C7—C12	1.433 (8)	C48—H48	0.9300
C8—C9	1.367 (9)	C49—H49	0.9300
C8—H8	0.9300	C50—C51	1.357 (9)
C9—C10	1.378 (8)	C50—H50	0.9300
C9—H9	0.9300	C51—H51	0.9300
C10—H10	0.9300	C52—C53	1.541 (8)
C11—C12	1.351 (9)	C52—H52A	0.9700
C11—H11	0.9300	C52—H52B	0.9700
C12—H12	0.9300	C53—C54	1.519 (8)
C13—C14	1.539 (8)	C53—H53A	0.9700
C13—H13A	0.9700	C53—H53B	0.9700
C13—H13B	0.9700	C54—H54A	0.9700
C14—C15	1.536 (8)	C54—H54B	0.9700
C14—H14A	0.9700	C55—C56	1.380 (8)
C14—H14B	0.9700	C55—C60	1.388 (9)
C15—H15A	0.9700	C56—C57	1.392 (10)
C15—H15B	0.9700	C56—H56	0.9300
C16—C17	1.363 (8)	C57—C58	1.367 (14)
C16—C21	1.379 (8)	C57—H57	0.9300
C17—C18	1.370 (9)	C58—C59	1.363 (13)
C17—H17	0.9300	C58—H58	0.9300
C18—C19	1.376 (10)	C59—C60	1.392 (10)
C18—H18	0.9300	C59—H59	0.9300
C19—C20	1.346 (9)	C60—H60	0.9300
C19—H19	0.9300	C61—C66	1.381 (8)
C20—C21	1.384 (8)	C61—C62	1.377 (8)
C20—H20	0.9300	C62—C63	1.379 (9)
C21—H21	0.9300	C62—H62	0.9300
C22—C23	1.361 (9)	C63—C64	1.375 (10)
C22—C27	1.372 (8)	C63—H63	0.9300
C23—C24	1.409 (11)	C64—C65	1.379 (10)
C23—H23	0.9300	C64—H64	0.9300
C24—C25	1.328 (12)	C65—C66	1.382 (8)
C24—H24	0.9300	C65—H65	0.9300
C25—C26	1.326 (12)	C66—H66	0.9300
C25—H25	0.9300	C67—C68	1.382 (8)
C26—C27	1.390 (10)	C67—C72	1.396 (8)
C26—H26	0.9300	C68—C69	1.394 (8)
C27—H27	0.9300	C68—H68	0.9300
C28—C29	1.369 (7)	C69—C70	1.352 (10)
C28—C33	1.397 (8)	C69—H69	0.9300
C29—C30	1.390 (9)	C70—C71	1.348 (10)
C29—H29	0.9300	C70—H70	0.9300
C30—C31	1.375 (10)	C71—C72	1.384 (9)
C30—H30	0.9300	C71—H71	0.9300
C31—C32	1.356 (10)	C72—H72	0.9300
C31—H31	0.9300	C73—C74	1.375 (8)
C32—C33	1.382 (9)	C73—C78	1.372 (9)
C32—H32	0.9300	C74—C75	1.411 (10)
C33—H33	0.9300	C74—H74	0.9300
C34—C39	1.393 (8)	C75—C76	1.375 (14)
C34—C35	1.400 (8)	C75—H75	0.9300
C35—C36	1.387 (8)	C76—C77	1.343 (13)
C35—H35	0.9300	C76—H76	0.9300
C36—C37	1.367 (10)	C77—C78	1.365 (10)
C36—H36	0.9300	C77—H77	0.9300
C37—C38	1.369 (10)	C78—H78	0.9300
C37—H37	0.9300	Cl1—O4	1.379 (6)
C38—C39	1.382 (9)	Cl1—O1	1.392 (5)
C38—H38	0.9300	Cl1—O3	1.401 (6)
C39—H39	0.9300	Cl1—O2	1.416 (6)
Cu2—N4	2.058 (4)	Cl2—O6	1.388 (6)
Cu2—N3	2.077 (4)	Cl2—O5	1.414 (5)
Cu2—P3	2.2302 (16)	Cl2—O8	1.406 (7)
Cu2—P4	2.2457 (17)	Cl2—O7	1.417 (5)
			
N2—Cu1—N1	81.93 (17)	C55—P3—C61	102.1 (3)
N2—Cu1—P2	117.00 (14)	C55—P3—C52	106.5 (3)
N1—Cu1—P2	119.79 (13)	C61—P3—C52	101.0 (2)
N2—Cu1—P1	124.23 (14)	C55—P3—Cu2	113.53 (18)
N1—Cu1—P1	108.49 (14)	C61—P3—Cu2	121.72 (19)
P2—Cu1—P1	104.68 (6)	C52—P3—Cu2	110.39 (18)
C16—P1—C22	103.0 (3)	C67—P4—C73	103.2 (3)
C16—P1—C13	103.0 (3)	C67—P4—C54	102.8 (3)
C22—P1—C13	103.7 (3)	C73—P4—C54	103.1 (3)
C16—P1—Cu1	121.58 (19)	C67—P4—Cu2	120.40 (19)
C22—P1—Cu1	115.1 (2)	C73—P4—Cu2	116.8 (2)
C13—P1—Cu1	108.6 (2)	C54—P4—Cu2	108.4 (2)
C34—P2—C28	102.7 (3)	C40—N3—C44	118.3 (4)
C34—P2—C15	101.0 (3)	C40—N3—Cu2	130.3 (4)
C28—P2—C15	105.9 (3)	C44—N3—Cu2	111.3 (3)
C34—P2—Cu1	120.14 (19)	C49—N4—C45	117.5 (4)
C28—P2—Cu1	115.02 (18)	C49—N4—Cu2	130.5 (4)
C15—P2—Cu1	110.3 (2)	C45—N4—Cu2	111.5 (3)
C1—N1—C5	116.4 (5)	N3—C40—C41	122.6 (5)
C1—N1—Cu1	131.6 (4)	N3—C40—H40	118.7
C5—N1—Cu1	111.4 (3)	C41—C40—H40	118.7
C10—N2—C6	116.0 (5)	C42—C41—C40	119.2 (5)
C10—N2—Cu1	132.1 (4)	C42—C41—H41	120.4
C6—N2—Cu1	111.9 (3)	C40—C41—H41	120.4
N1—C1—C2	124.8 (5)	C41—C42—C43	120.3 (5)
N1—C1—H1	117.6	C41—C42—H42	119.8
C2—C1—H1	117.6	C43—C42—H42	119.8
C3—C2—C1	119.3 (5)	C44—C43—C42	116.8 (5)
C3—C2—H2	120.4	C44—C43—C50	119.5 (5)
C1—C2—H2	120.4	C42—C43—C50	123.7 (5)
C2—C3—C4	119.7 (5)	N3—C44—C43	122.8 (5)
C2—C3—H3	120.2	N3—C44—C45	117.6 (4)
C4—C3—H3	120.2	C43—C44—C45	119.6 (5)
C5—C4—C3	116.5 (5)	N4—C45—C46	122.6 (5)
C5—C4—C11	120.1 (6)	N4—C45—C44	117.9 (4)
C3—C4—C11	123.4 (6)	C46—C45—C44	119.4 (5)
N1—C5—C4	123.3 (5)	C45—C46—C47	117.2 (5)
N1—C5—C6	117.1 (4)	C45—C46—C51	119.3 (5)
C4—C5—C6	119.6 (5)	C47—C46—C51	123.5 (5)
N2—C6—C7	123.5 (5)	C48—C47—C46	119.1 (5)
N2—C6—C5	117.4 (4)	C48—C47—H47	120.4
C7—C6—C5	119.0 (5)	C46—C47—H47	120.4
C8—C7—C6	117.1 (5)	C47—C48—C49	120.6 (6)
C8—C7—C12	123.7 (5)	C47—C48—H48	119.7
C6—C7—C12	119.2 (5)	C49—C48—H48	119.7
C9—C8—C7	120.1 (5)	N4—C49—C48	122.9 (5)
C9—C8—H8	120.0	N4—C49—H49	118.5
C7—C8—H8	120.0	C48—C49—H49	118.5
C8—C9—C10	118.2 (5)	C51—C50—C43	120.8 (6)
C8—C9—H9	120.9	C51—C50—H50	119.6
C10—C9—H9	120.9	C43—C50—H50	119.6
N2—C10—C9	125.1 (6)	C50—C51—C46	121.2 (6)
N2—C10—H10	117.5	C50—C51—H51	119.4
C9—C10—H10	117.5	C46—C51—H51	119.4
C12—C11—C4	120.1 (6)	C53—C52—P3	116.8 (4)
C12—C11—H11	119.9	C53—C52—H52A	108.1
C4—C11—H11	119.9	P3—C52—H52A	108.1
C11—C12—C7	121.9 (6)	C53—C52—H52B	108.1
C11—C12—H12	119.1	P3—C52—H52B	108.1
C7—C12—H12	119.1	H52A—C52—H52B	107.3
C14—C13—P1	112.9 (4)	C54—C53—C52	115.1 (5)
C14—C13—H13A	109.0	C54—C53—H53A	108.5
P1—C13—H13A	109.0	C52—C53—H53A	108.5
C14—C13—H13B	109.0	C54—C53—H53B	108.5
P1—C13—H13B	109.0	C52—C53—H53B	108.5
H13A—C13—H13B	107.8	H53A—C53—H53B	107.5
C13—C14—C15	115.2 (5)	C53—C54—P4	112.9 (4)
C13—C14—H14A	108.5	C53—C54—H54A	109.0
C15—C14—H14A	108.5	P4—C54—H54A	109.0
C13—C14—H14B	108.5	C53—C54—H54B	109.0
C15—C14—H14B	108.5	P4—C54—H54B	109.0
H14A—C14—H14B	107.5	H54A—C54—H54B	107.8
C14—C15—P2	115.6 (4)	C56—C55—C60	119.4 (6)
C14—C15—H15A	108.4	C56—C55—P3	123.6 (5)
P2—C15—H15A	108.4	C60—C55—P3	117.0 (5)
C14—C15—H15B	108.4	C55—C56—C57	120.1 (8)
P2—C15—H15B	108.4	C55—C56—H56	119.9
H15A—C15—H15B	107.5	C57—C56—H56	119.9
C17—C16—C21	118.1 (5)	C58—C57—C56	119.8 (8)
C17—C16—P1	123.3 (4)	C58—C57—H57	120.1
C21—C16—P1	118.6 (5)	C56—C57—H57	120.1
C16—C17—C18	121.8 (6)	C59—C58—C57	120.9 (8)
C16—C17—H17	119.1	C59—C58—H58	119.5
C18—C17—H17	119.1	C57—C58—H58	119.5
C17—C18—C19	119.6 (7)	C58—C59—C60	119.9 (9)
C17—C18—H18	120.2	C58—C59—H59	120.0
C19—C18—H18	120.2	C60—C59—H59	120.0
C20—C19—C18	119.2 (6)	C55—C60—C59	119.9 (7)
C20—C19—H19	120.4	C55—C60—H60	120.1
C18—C19—H19	120.4	C59—C60—H60	120.1
C19—C20—C21	121.2 (6)	C66—C61—C62	118.1 (6)
C19—C20—H20	119.4	C66—C61—P3	119.7 (5)
C21—C20—H20	119.4	C62—C61—P3	122.1 (5)
C20—C21—C16	119.9 (6)	C61—C62—C63	121.7 (6)
C20—C21—H21	120.0	C61—C62—H62	119.1
C16—C21—H21	120.0	C63—C62—H62	119.1
C23—C22—C27	117.9 (6)	C64—C63—C62	119.5 (7)
C23—C22—P1	123.8 (5)	C64—C63—H63	120.3
C27—C22—P1	118.2 (5)	C62—C63—H63	120.3
C22—C23—C24	118.8 (8)	C63—C64—C65	119.9 (7)
C22—C23—H23	120.6	C63—C64—H64	120.1
C24—C23—H23	120.6	C65—C64—H64	120.1
C25—C24—C23	122.9 (9)	C66—C65—C64	119.9 (7)
C25—C24—H24	118.6	C66—C65—H65	120.1
C23—C24—H24	118.6	C64—C65—H65	120.1
C24—C25—C26	118.2 (8)	C61—C66—C65	121.0 (6)
C24—C25—H25	120.9	C61—C66—H66	119.5
C26—C25—H25	120.9	C65—C66—H66	119.5
C25—C26—C27	121.6 (8)	C68—C67—C72	117.2 (5)
C25—C26—H26	119.2	C68—C67—P4	119.9 (5)
C27—C26—H26	119.2	C72—C67—P4	122.9 (5)
C22—C27—C26	120.7 (7)	C69—C68—C67	121.5 (6)
C22—C27—H27	119.7	C69—C68—H68	119.3
C26—C27—H27	119.7	C67—C68—H68	119.3
C29—C28—C33	118.7 (6)	C70—C69—C68	119.0 (6)
C29—C28—P2	124.0 (5)	C70—C69—H69	120.5
C33—C28—P2	117.3 (4)	C68—C69—H69	120.5
C28—C29—C30	119.7 (6)	C69—C70—C71	121.6 (7)
C28—C29—H29	120.2	C69—C70—H70	119.2
C30—C29—H29	120.2	C71—C70—H70	119.2
C31—C30—C29	121.4 (6)	C70—C71—C72	120.0 (7)
C31—C30—H30	119.3	C70—C71—H71	120.0
C29—C30—H30	119.3	C72—C71—H71	120.0
C32—C31—C30	118.9 (7)	C71—C72—C67	120.7 (6)
C32—C31—H31	120.5	C71—C72—H72	119.6
C30—C31—H31	120.5	C67—C72—H72	119.6
C31—C32—C33	120.7 (7)	C74—C73—C78	116.9 (6)
C31—C32—H32	119.6	C74—C73—P4	118.6 (5)
C33—C32—H32	119.6	C78—C73—P4	124.5 (5)
C32—C33—C28	120.5 (6)	C73—C74—C75	120.8 (8)
C32—C33—H33	119.7	C73—C74—H74	119.6
C28—C33—H33	119.7	C75—C74—H74	119.6
C39—C34—C35	117.8 (5)	C76—C75—C74	119.5 (9)
C39—C34—P2	123.7 (4)	C76—C75—H75	120.2
C35—C34—P2	118.4 (5)	C74—C75—H75	120.2
C36—C35—C34	121.1 (7)	C77—C76—C75	119.3 (9)
C36—C35—H35	119.5	C77—C76—H76	120.3
C34—C35—H35	119.5	C75—C76—H76	120.3
C37—C36—C35	119.6 (6)	C76—C77—C78	120.9 (10)
C37—C36—H36	120.2	C76—C77—H77	119.6
C35—C36—H36	120.2	C78—C77—H77	119.6
C36—C37—C38	120.4 (6)	C77—C78—C73	122.5 (8)
C36—C37—H37	119.8	C77—C78—H78	118.7
C38—C37—H37	119.8	C73—C78—H78	118.7
C37—C38—C39	120.7 (7)	O4—Cl1—O1	110.8 (5)
C37—C38—H38	119.6	O4—Cl1—O3	111.5 (5)
C39—C38—H38	119.6	O1—Cl1—O3	109.2 (3)
C38—C39—C34	120.3 (6)	O4—Cl1—O2	107.8 (5)
C38—C39—H39	119.8	O1—Cl1—O2	108.1 (4)
C34—C39—H39	119.8	O3—Cl1—O2	109.4 (5)
N4—Cu2—N3	81.48 (16)	O6—Cl2—O5	110.7 (4)
N4—Cu2—P3	119.94 (13)	O6—Cl2—O8	108.8 (5)
N3—Cu2—P3	116.47 (13)	O5—Cl2—O8	108.1 (4)
N4—Cu2—P4	111.65 (14)	O6—Cl2—O7	111.0 (4)
N3—Cu2—P4	122.40 (13)	O5—Cl2—O7	110.7 (3)
P3—Cu2—P4	104.56 (6)	O8—Cl2—O7	107.5 (4)
			
N2—Cu1—P1—C16	46.7 (3)	N4—Cu2—P3—C55	−135.9 (3)
N1—Cu1—P1—C16	139.5 (2)	N3—Cu2—P3—C55	−40.4 (3)
P2—Cu1—P1—C16	−91.6 (2)	P4—Cu2—P3—C55	98.0 (2)
N2—Cu1—P1—C22	−78.7 (2)	N4—Cu2—P3—C61	−13.3 (3)
N1—Cu1—P1—C22	14.1 (2)	N3—Cu2—P3—C61	82.2 (3)
P2—Cu1—P1—C22	143.1 (2)	P4—Cu2—P3—C61	−139.4 (2)
N2—Cu1—P1—C13	165.7 (2)	N4—Cu2—P3—C52	104.6 (2)
N1—Cu1—P1—C13	−101.5 (2)	N3—Cu2—P3—C52	−159.9 (2)
P2—Cu1—P1—C13	27.4 (2)	P4—Cu2—P3—C52	−21.5 (2)
N2—Cu1—P2—C34	78.5 (3)	N4—Cu2—P4—C67	138.2 (2)
N1—Cu1—P2—C34	−17.8 (3)	N3—Cu2—P4—C67	44.6 (2)
P1—Cu1—P2—C34	−139.7 (2)	P3—Cu2—P4—C67	−90.7 (2)
N2—Cu1—P2—C28	−45.0 (2)	N4—Cu2—P4—C73	11.8 (2)
N1—Cu1—P2—C28	−141.3 (2)	N3—Cu2—P4—C73	−81.8 (2)
P1—Cu1—P2—C28	96.8 (2)	P3—Cu2—P4—C73	142.9 (2)
N2—Cu1—P2—C15	−164.7 (2)	N4—Cu2—P4—C54	−104.1 (2)
N1—Cu1—P2—C15	99.0 (2)	N3—Cu2—P4—C54	162.3 (2)
P1—Cu1—P2—C15	−22.9 (2)	P3—Cu2—P4—C54	27.1 (2)
N2—Cu1—N1—C1	173.6 (6)	N4—Cu2—N3—C40	178.7 (5)
P2—Cu1—N1—C1	−69.8 (6)	P3—Cu2—N3—C40	59.5 (5)
P1—Cu1—N1—C1	50.2 (5)	P4—Cu2—N3—C40	−71.0 (5)
N2—Cu1—N1—C5	2.9 (4)	N4—Cu2—N3—C44	−2.6 (4)
P2—Cu1—N1—C5	119.5 (3)	P3—Cu2—N3—C44	−121.9 (3)
P1—Cu1—N1—C5	−120.6 (3)	P4—Cu2—N3—C44	107.7 (3)
N1—Cu1—N2—C10	−179.4 (6)	N3—Cu2—N4—C49	175.9 (5)
P2—Cu1—N2—C10	61.2 (6)	P3—Cu2—N4—C49	−68.3 (5)
P1—Cu1—N2—C10	−72.5 (6)	P4—Cu2—N4—C49	54.4 (5)
N1—Cu1—N2—C6	−0.3 (4)	N3—Cu2—N4—C45	4.2 (4)
P2—Cu1—N2—C6	−119.7 (3)	P3—Cu2—N4—C45	119.9 (3)
P1—Cu1—N2—C6	106.6 (3)	P4—Cu2—N4—C45	−117.4 (3)
C5—N1—C1—C2	−0.5 (9)	C44—N3—C40—C41	0.6 (8)
Cu1—N1—C1—C2	−170.9 (5)	Cu2—N3—C40—C41	179.3 (4)
N1—C1—C2—C3	1.2 (10)	N3—C40—C41—C42	−0.6 (9)
C1—C2—C3—C4	−2.9 (10)	C40—C41—C42—C43	0.0 (9)
C2—C3—C4—C5	3.8 (9)	C41—C42—C43—C44	0.6 (9)
C2—C3—C4—C11	−176.6 (6)	C41—C42—C43—C50	−178.4 (6)
C1—N1—C5—C4	1.6 (8)	C40—N3—C44—C43	0.0 (8)
Cu1—N1—C5—C4	173.9 (4)	Cu2—N3—C44—C43	−178.9 (4)
C1—N1—C5—C6	−177.3 (5)	C40—N3—C44—C45	179.5 (5)
Cu1—N1—C5—C6	−5.0 (6)	Cu2—N3—C44—C45	0.6 (6)
C3—C4—C5—N1	−3.3 (9)	C42—C43—C44—N3	−0.5 (8)
C11—C4—C5—N1	177.1 (6)	C50—C43—C44—N3	178.4 (5)
C3—C4—C5—C6	175.7 (5)	C42—C43—C44—C45	180.0 (5)
C11—C4—C5—C6	−4.0 (9)	C50—C43—C44—C45	−1.1 (8)
C10—N2—C6—C7	−0.4 (8)	C49—N4—C45—C46	0.7 (8)
Cu1—N2—C6—C7	−179.7 (4)	Cu2—N4—C45—C46	173.6 (4)
C10—N2—C6—C5	177.0 (5)	C49—N4—C45—C44	−178.2 (5)
Cu1—N2—C6—C5	−2.3 (6)	Cu2—N4—C45—C44	−5.2 (6)
N1—C5—C6—N2	5.1 (7)	N3—C44—C45—N4	3.2 (7)
C4—C5—C6—N2	−173.9 (5)	C43—C44—C45—N4	−177.3 (5)
N1—C5—C6—C7	−177.4 (5)	N3—C44—C45—C46	−175.7 (5)
C4—C5—C6—C7	3.6 (8)	C43—C44—C45—C46	3.8 (8)
N2—C6—C7—C8	−2.1 (8)	N4—C45—C46—C47	−1.8 (8)
C5—C6—C7—C8	−179.4 (5)	C44—C45—C46—C47	177.0 (5)
N2—C6—C7—C12	176.8 (5)	N4—C45—C46—C51	178.1 (5)
C5—C6—C7—C12	−0.6 (8)	C44—C45—C46—C51	−3.1 (8)
C6—C7—C8—C9	2.8 (9)	C45—C46—C47—C48	2.1 (9)
C12—C7—C8—C9	−176.0 (6)	C51—C46—C47—C48	−177.8 (6)
C7—C8—C9—C10	−1.0 (9)	C46—C47—C48—C49	−1.4 (10)
C6—N2—C10—C9	2.4 (9)	C45—N4—C49—C48	0.2 (9)
Cu1—N2—C10—C9	−178.4 (5)	Cu2—N4—C49—C48	−171.2 (5)
C8—C9—C10—N2	−1.8 (10)	C47—C48—C49—N4	0.2 (10)
C5—C4—C11—C12	1.2 (10)	C44—C43—C50—C51	−2.4 (9)
C3—C4—C11—C12	−178.4 (7)	C42—C43—C50—C51	176.4 (6)
C4—C11—C12—C7	2.0 (11)	C43—C50—C51—C46	3.2 (10)
C8—C7—C12—C11	176.5 (7)	C45—C46—C51—C50	−0.4 (9)
C6—C7—C12—C11	−2.3 (10)	C47—C46—C51—C50	179.5 (7)
C16—P1—C13—C14	76.4 (5)	C55—P3—C52—C53	−82.1 (5)
C22—P1—C13—C14	−176.5 (4)	C61—P3—C52—C53	171.6 (4)
Cu1—P1—C13—C14	−53.7 (4)	Cu2—P3—C52—C53	41.5 (5)
P1—C13—C14—C15	82.3 (5)	P3—C52—C53—C54	−74.9 (6)
C13—C14—C15—P2	−76.0 (6)	C52—C53—C54—P4	82.3 (6)
C34—P2—C15—C14	171.4 (4)	C67—P4—C54—C53	74.0 (5)
C28—P2—C15—C14	−81.8 (5)	C73—P4—C54—C53	−179.0 (4)
Cu1—P2—C15—C14	43.3 (5)	Cu2—P4—C54—C53	−54.5 (4)
C22—P1—C16—C17	−64.1 (6)	C61—P3—C55—C56	106.5 (6)
C13—P1—C16—C17	43.5 (6)	C52—P3—C55—C56	1.0 (6)
Cu1—P1—C16—C17	165.2 (5)	Cu2—P3—C55—C56	−120.7 (5)
C22—P1—C16—C21	115.1 (5)	C61—P3—C55—C60	−73.1 (5)
C13—P1—C16—C21	−137.3 (5)	C52—P3—C55—C60	−178.5 (5)
Cu1—P1—C16—C21	−15.6 (6)	Cu2—P3—C55—C60	59.8 (5)
C21—C16—C17—C18	0.4 (10)	C60—C55—C56—C57	−0.7 (10)
P1—C16—C17—C18	179.6 (6)	P3—C55—C56—C57	179.7 (6)
C16—C17—C18—C19	2.1 (12)	C55—C56—C57—C58	−0.3 (12)
C17—C18—C19—C20	−3.6 (12)	C56—C57—C58—C59	1.2 (14)
C18—C19—C20—C21	2.6 (11)	C57—C58—C59—C60	−1.2 (14)
C19—C20—C21—C16	−0.1 (11)	C56—C55—C60—C59	0.8 (10)
C17—C16—C21—C20	−1.4 (9)	P3—C55—C60—C59	−179.6 (6)
P1—C16—C21—C20	179.4 (5)	C58—C59—C60—C55	0.2 (12)
C16—P1—C22—C23	80.0 (6)	C55—P3—C61—C66	154.9 (5)
C13—P1—C22—C23	−27.1 (7)	C52—P3—C61—C66	−95.3 (5)
Cu1—P1—C22—C23	−145.5 (6)	Cu2—P3—C61—C66	27.1 (5)
C16—P1—C22—C27	−102.0 (5)	C55—P3—C61—C62	−29.3 (5)
C13—P1—C22—C27	150.9 (5)	C52—P3—C61—C62	80.5 (5)
Cu1—P1—C22—C27	32.5 (5)	Cu2—P3—C61—C62	−157.0 (4)
C27—C22—C23—C24	0.0 (11)	C66—C61—C62—C63	1.2 (9)
P1—C22—C23—C24	178.1 (7)	P3—C61—C62—C63	−174.7 (5)
C22—C23—C24—C25	0.1 (15)	C61—C62—C63—C64	−0.7 (10)
C23—C24—C25—C26	0.6 (16)	C62—C63—C64—C65	−0.4 (10)
C24—C25—C26—C27	−1.3 (13)	C63—C64—C65—C66	0.8 (10)
C23—C22—C27—C26	−0.7 (9)	C62—C61—C66—C65	−0.7 (9)
P1—C22—C27—C26	−178.9 (5)	P3—C61—C66—C65	175.3 (5)
C25—C26—C27—C22	1.4 (11)	C64—C65—C66—C61	−0.3 (9)
C34—P2—C28—C29	107.9 (5)	C73—P4—C67—C68	112.9 (5)
C15—P2—C28—C29	2.3 (6)	C54—P4—C67—C68	−140.1 (5)
Cu1—P2—C28—C29	−119.8 (5)	Cu2—P4—C67—C68	−19.6 (5)
C34—P2—C28—C33	−70.6 (5)	C73—P4—C67—C72	−65.6 (6)
C15—P2—C28—C33	−176.1 (5)	C54—P4—C67—C72	41.4 (6)
Cu1—P2—C28—C33	61.8 (5)	Cu2—P4—C67—C72	162.0 (5)
C33—C28—C29—C30	0.0 (9)	C72—C67—C68—C69	−1.7 (9)
P2—C28—C29—C30	−178.4 (5)	P4—C67—C68—C69	179.7 (5)
C28—C29—C30—C31	1.6 (11)	C67—C68—C69—C70	1.3 (10)
C29—C30—C31—C32	−1.6 (12)	C68—C69—C70—C71	−0.9 (11)
C30—C31—C32—C33	0.2 (13)	C69—C70—C71—C72	1.1 (13)
C31—C32—C33—C28	1.3 (13)	C70—C71—C72—C67	−1.6 (12)
C29—C28—C33—C32	−1.4 (10)	C68—C67—C72—C71	1.9 (10)
P2—C28—C33—C32	177.2 (6)	P4—C67—C72—C71	−179.7 (6)
C28—P2—C34—C39	−21.5 (5)	C67—P4—C73—C74	−109.9 (5)
C15—P2—C34—C39	87.7 (5)	C54—P4—C73—C74	143.4 (5)
Cu1—P2—C34—C39	−150.8 (4)	Cu2—P4—C73—C74	24.7 (5)
C28—P2—C34—C35	159.0 (4)	C67—P4—C73—C78	70.6 (6)
C15—P2—C34—C35	−91.8 (5)	C54—P4—C73—C78	−36.2 (6)
Cu1—P2—C34—C35	29.7 (5)	Cu2—P4—C73—C78	−154.9 (5)
C39—C34—C35—C36	−1.5 (9)	C78—C73—C74—C75	1.3 (10)
P2—C34—C35—C36	178.0 (5)	P4—C73—C74—C75	−178.3 (5)
C34—C35—C36—C37	0.0 (10)	C73—C74—C75—C76	−1.2 (12)
C35—C36—C37—C38	1.8 (10)	C74—C75—C76—C77	−0.6 (14)
C36—C37—C38—C39	−2.1 (11)	C75—C76—C77—C78	2.3 (15)
C37—C38—C39—C34	0.6 (10)	C76—C77—C78—C73	−2.3 (14)
C35—C34—C39—C38	1.2 (9)	C74—C73—C78—C77	0.4 (11)
P2—C34—C39—C38	−178.3 (5)	P4—C73—C78—C77	−180.0 (6)

## References

[bb1] Abakumov, G. A., Cherkasov, V. K., Krashilina, A. V., Eremenko, I. L. & Nefedov, S. E. (1998). *Russ. Chem. Bull.* **11**, 2333–2340.

[bb2] Affandi, D., Price, S. J. B., Effendy, H., Harvey, P. J., Healy, P. C., Ruch, B. E. & White, A. H. (1997). *J. Chem. Soc. Dalton Trans.* pp. 1411–1420.

[bb3] Alanidis, P., Cox, P. J., Divanidis, S. & Tsipis, A. C. (2002). *Inorg. Chem.* **41**, 6875–6886. 10.1021/ic025896i 12470086

[bb4] Bruker (2007). *SMART* and *SAINT* Bruker AXS Inc., Madison, Wisconsin, USA.

[bb5] Farrugia, L. J. (1999). *J. Appl. Cryst.* **32**, 837–838.

[bb6] Flack, H. D. (1983). *Acta Cryst.* A**39**, 876–881.

[bb7] Fournier, E., Sicard, S., Decken, A. & Harvey, P. D. (2004). *Inorg. Chem.* **43**, 1491–1501. 10.1021/ic034780z 14966987

[bb8] Jin, Q. H., Chen, L. M., Li, P. Z., Deng, S. F. & Wang, R. (2009). *Inorg. Chim. Acta*, **362**, 5224–5230.

[bb9] Macrae, C. F., Bruno, I. J., Chisholm, J. A., Edgington, P. R., McCabe, P., Pidcock, E., Rodriguez-Monge, L., Taylor, R., van de Streek, J. & Wood, P. A. (2008). *J. Appl. Cryst.* **41**, 466–470.

[bb10] Parson, S. & Gould, B. (2001). *ROTAX* University of Edinburgh, Scotland.

[bb11] Saito, K., Arai, T., Takahashi, N., Tsukuda, T. & Tsubomura, T. (2006). *Dalton Trans.* pp. 4444–4448. 10.1039/b608641a 16981018

[bb12] Sheldrick, G. M. (1996). *SADABS* University of Göttingen, Germany.

[bb13] Sheldrick, G. M. (2008). *Acta Cryst.* A**64**, 112–122. 10.1107/S0108767307043930 18156677

[bb14] Spek, A. L. (2009). *Acta Cryst.* D**65**, 148–155. 10.1107/S090744490804362X PMC2631630 19171970

